# Psychometric evaluation of the Persian version of the Rosenberg Self-Esteem Scale among medical sciences students

**DOI:** 10.3389/fpsyg.2024.1451862

**Published:** 2024-11-12

**Authors:** Ali Abbasi, Ahmadreza Moradkhani, Bahar Shahri, Hamed Khosravi, Atena Sohrabi, Seyedmohammad Mirhosseini

**Affiliations:** ^1^Department of Nursing, School of Nursing and Midwifery, Shahroud University of Medical Sciences, Shahroud, Iran; ^2^Student Research Committee, School of Allied Medical Sciences, Shahroud University of Medical Sciences, Shahroud, Iran; ^3^Nursing Care Research Center, Nursing and Midwifery Faculty, Semnan University of Medical Sciences, Semnan, Iran; ^4^Student Research Committee, Faculty of Nursing and Midwifery, Semnan University of Medical Sciences, Semnan, Iran

**Keywords:** self-esteem, scale, validity, reliability, psychometric

## Abstract

**Introduction:**

The present research carried out to assess the psychometric properties of the Persian version of the Rosenberg Self-Esteem Scale (RSES) among medical sciences students.

**Methods:**

This methodological study took place at Shahroud and Semnan Universities of Medical Sciences in Iran. A sample of 380 medical sciences students was selected through convenience sampling. The study assessed face and content validity, and maximum likelihood explanatory factor analysis (MLEFA) was performed. To evaluate the proposed model by MLEFA, confirmatory factor analysis was carried out. Reliability was examined using Cronbach’s alpha coefficient, McDonald’s omega, and the intra-class correlation coefficient.

**Results:**

The students had an average age of 22.49 ± 2.72 years. The maximum likelihood explanatory factor analysis (MLEFA) divided the RSES into two components: positive self-esteem and negative self-esteem, which together explained 50.7% of the total variance. Confirmatory factor analysis showed that the model fit the observed data well. The resulting subscales exhibited high internal consistency and stability.

**Conclusion:**

The findings of the current study indicate that the Persian version of the Rosenberg Self-Esteem Scale possesses acceptable validity and reliability among Iranian medical sciences students.

## 1 Introduction

Medical sciences students often face challenges such as handling stress in clinical environments and dealing with patients’ problems, putting them at risk of losing their mental health (Jaafari et al., 2021). In recent years, self-esteem, a fundamental component of mental health, has garnered attention among medical sciences students (Valizadeh et al., 2016). It is worth noting that self-esteem, recognized as one of the crucial psychological aspects these students, can significantly influence various aspects of their lives, particularly academic success (Neroni et al., 2022), academic self-efficacy (Luo et al., 2022), and the manifestation of negative psychological symptoms such as depression (Mirhosseini et al., 2022). Self-esteem is defined as a feeling of self-worth and self-respect that plays a crucial role in individuals’ comfort and well-being (Monteiro et al., 2022; Gnambs et al., 2018; Coopersmith, 1990). Coopersmith (1990) described self-esteem as an evaluation a person makes about themselves, which is typically maintained over time. Self-esteem reflects an attitude of approval or disapproval (Rosenberg, 1965). Rosenberg similarly defined self-esteem as an individual’s overall feeling of self-worth, which can be either negative or positive, derived from one’s evaluation of their own characteristics (Johan et al., 2017). Negative self-esteem (NSE) is significant in psychopathology and can lead to a lack of self-confidence, self-hatred, and pessimism (Billa et al., 2023). In contrast, individuals with Positive Self-Esteem (PSE) recognize their worth and take pride in their abilities, skills, and achievements. Consequently, their self-esteem grows with each successful experience and interaction (Pullmann and Allik, 2000).

High self-esteem is the belief that one deserves the privilege and admiration of others due to being unique, special, and having fantasies of brilliance and beauty. In the context of university students, high self-esteem is not solely responsible for academic success and achievement but is often a result of these successes. It also partially contributes to success and helps facilitate persistence after failure (Baumeister et al., 2003). Conversely, low self-esteem involves a conflict between competing aspects of the self, such as the real self versus the ideal self. Simply put, it reflects a significant gap between how individuals perceive their current selves and the ideal selves they aspire to be within their value system. This disparity can lead to psychosocial weaknesses and a lack of self-confidence, resulting in problems and risky behaviors (Mann et al., 2004). Low self-esteem is a risk factor for symptoms of depression and anxiety, eating disorders, violence, and substance abuse among medical sciences students (Younes et al., 2016; Mallaram et al., 2023). According to the results of a qualitative study, in Iranian culture, the self-esteem of medical sciences students, particularly nursing students, is closely tied to their sense of competence. Their self-esteem is shaped by their perceived level of professionalism, socialization within the profession, and their enthusiasm for their role. Consequently, when students take pride in their role, they tend to enjoy their academic experience and all that it encompasses, including interactions with colleagues, performing tasks, and their overall work (Zamanzadeh et al., 2016).

Among the common tools for measuring self-esteem are Rosenberg’s Self-Esteem Scale (Rosenberg, 1965) and Coopersmith’s Self-Esteem Inventory (Coopersmith, 1990). Initially, Rosenberg’s scale was developed to assess feelings of acceptance and overall self-worth in teenagers, while Coopersmith’s inventory was designed to evaluate attitudes toward oneself in specific areas such as relationships with parents, peers, school, and personal interests. However, compared to Coopersmith’s inventory, Rosenberg’s Self-Esteem Scale is simpler and shorter, with appropriate reliability and validity. It is versatile enough to be used with any age group possessing an elementary level of education (Mayordomo et al., 2020; Butler and Gasson, 2005).

This 10-item scale is widely recognized as the most commonly used tool for measuring self-esteem. It has been translated and validated in numerous societies around the world (Tinakon and Nahathai, 2012; Gómez-Lugo et al., 2016; Jiang et al., 2023; Moksnes et al., 2024; Aachal and Translation, 2024). Kielkiewicz et al. (2020) examined the construct validity and dimensions of Rosenberg’s Self-Esteem Scale and its relationship with spiritual values in the Irish population. In the study by Tulachan et al. (2022), the validity and reliability of this scale were investigated in the Nepalese adult population. Mayordomo et al. (2020) conducted a study aiming to adapt and validate the Rosenberg Self-Esteem Scale in the Spanish elderly population, based on the initial single-factor model provided by the creator of the scale (Mayordomo et al., 2020). According to its creator, this tool is a single-factor scale (Rosenberg, 1965). However, in recent years, emphasis has been placed on the existence of two factors: positive and negative self-esteem. Researchers using factor analysis have demonstrated that this scale has a two-factor structure comprising positive and negative self-images. Five items with positive wording are grouped under “Positive Self-Esteem” (PSE), while five items with negative wording are grouped under “Negative Self-Esteem” (NPS) (Gómez-Lugo et al., 2016; Fromont et al., 2017; Michaelides et al., 2016). Various studies have also shown that the single-factor model of this scale has a poor fit when analyzed using confirmatory factor analysis. They found that the two-factor model, with distinct positive and negative self-images, provides a better fit (Supple et al., 2013).

In Iran, studies have been conducted on the psychometrics of the Persian version of this scale. Rajabi and Karjo (2012) investigated the confirmatory structure of the two-factor model of the Persian version of the Rosenberg Self-Esteem Scale in Iranian students. The results showed that the fit indices in the two-factor model were better than those in the one-factor model. However, this study had a small sample size, and exploratory factor analysis was not performed, despite the tool being examined in a new population (Rajabi and Karjo, 2012). Exploratory factor analysis should be conducted for psychometric scales related to behavioral measures in different cultures (Arafat et al., 2016). Additionally, Joshanloo and Ghaedi (2008) assessed the validity and reliability of the Rosenberg Self-Esteem Scale in Iranian students. Their results from exploratory and confirmatory factor analyses supported a two-dimensional structure, encompassing self-liking and lack of self-derogation (Joshanloo and Ghaedi, 2008). It’s important to remember that changes in behavioral constructs like self-esteem are influenced by societal norms and values (Du et al., 2023; Reitz, 2022). Therefore, it is necessary to reevaluate the psychometric properties of this scale after several years since the aforementioned studies were conducted in Iran.

Based on the aforementioned studies, it can be said that the Rosenberg Self-Esteem Scale is the most widely used tool for measuring self-esteem. Despite the studies conducted on the psychometrics of the Persian version of this scale, many research gaps remain. Therefore, this study was conducted with the aim of determining the psychometric properties of the Rosenberg Self-Esteem Scale (RSES) in a population of Iranian medical sciences students.

## 2 Materials and methods

The current methodological study took place among medical sciences students at Shahroud and Semnan Universities of Medical Sciences from January to March 2024.

### 2.1 Scale

RSES was introduced in 1965 by Rosenberg, this scale aims to gage feelings of acceptance and overall self-worth. The RSES comprises 10 items and is presented as a single-factor scale according to the manufacturer’s specifications. Each item on the scale is scored on a spectrum of four options: “completely agree” a score of 3, “agree” a score of 2, “disagree” a score of 1, and “totally disagree” a score of zero. Consequently, the lowest and highest achievable scores on this scale are 0 and 30, respectively. A higher score indicates a greater level of self-esteem in the individual (Rosenberg, 1965).

### 2.2 Translation

Following email correspondence and obtaining permission to translate the tool, it was translated in line with the World Health Organization’s translation protocol, utilizing the forward-backward method. Initially, two separate translators translated the scale from English to Persian. Subsequently, a combined translation was derived from the two translations, selecting the best options. This combined version was then translated back into English by two translation experts and compared with the original English version by the research team (Sartorius and Kuyken, 1994). All suggestions were incorporated into the final version of the scale.

### 2.3 Face validity

Face validity assessment employed both qualitative and quantitative methods. Initially, qualitative face validity was gaged through face-to-face interviews with 10 medical sciences students, assessing appropriateness, difficulty, relevance, and ambiguity of the items. Subsequently, quantitative face validity was evaluated by 10 medical sciences students, who rated the importance of each item on a 5-point Likert scale (ranging from very important = 5 to not important = 1). The impact score for each item was calculated using the formula: Impact score = Frequency (%) * Importance. Frequency represents the percentage of respondents awarding 4 or 5 points to the item, while Importance signifies the average importance score based on the Likert scale. A threshold of 1.5 was determined based on an average score of 3 and a frequency of 50%. Items with an Impact score exceeding 1.5 were deemed suitable for further analysis and retained. Notably, items with an Impact score below 1.5 were not discarded but rather reviewed and modified as necessary (Sharif Nia et al., 2020).

### 2.4 Content validity

Similar to the preceding section, content validity assessment utilized both qualitative and quantitative methodologies. Qualitative assessment involved interviews with 12 experts comprising five psychologists, five nursing specialists, and two psychometric scale experts. These interviews aimed to evaluate item placement, language proficiency, and scale scoring accuracy in the Persian version. Based on expert feedback, necessary revisions were made to the items. Quantitative evaluation of content validity was conducted by computing the Content Validity Ratio (CVR) and Content Validity Index (CVI). The expert panel rated the importance of each item based on its necessity (1: not necessary, 2: useful but not necessary, 3: necessary). CVR was then calculated using the following formula:


$$CVR=\left(ne-\left[N/2\right]\right)/\left(N/2\right)\text{.}$$

In this formula, *N* represents the total number of experts in the panel, while ne signifies the number of experts who deemed the item as “necessary.” According to the Lawshe table, with a panel of 12 experts, the minimum acceptable Content Validity Ratio (CVR) was set at 0.56 (Lawshe, 1975).

Furthermore, the Content Validity Index (CVI) gaged the degree of relevance of scale items to the overall concept of the scale, as per expert opinions. Each item’s relevance was assessed and scored on a scale of “not relevant = 1,” “somewhat relevant = 2,” “relevant but needs revision = 3,” and “completely relevant = 4.” CVI for each item was calculated by dividing the number of experts who rated the item 3 or 4 by the total number of experts. An acceptable CVI at this stage was considered to be above 0.79, indicating adequacy. Values falling between 0.79 and 0.70 were deemed questionable, necessitating revision, while items with values below 0.70 were deemed unacceptable and removed. The Scale Content Validity Index (S-CVI) and Scale Content Validity Ratio (S-CVR) were derived by averaging the individual CVI and CVR values, respectively. A minimum acceptable S-CVI value of 0.9 was set (Hyrkäs et al., 2003). Additionally, the modified Kappa statistic was calculated for each item to assess chance agreement among the expert panel, with items scoring 0.7 or higher considered appropriate (Wynd et al., 2003).

### 2.5 Participants and the study setting

To fulfill the objectives of this study and carry out both Exploratory Factor Analysis (EFA) and Confirmatory Factor Analysis (CFA), following Munro’s recommendations, 5–10 medical sciences students were chosen for each item (Munro, 2005). A total of 380 medical sciences students from Shahroud and Semnan Universities of Medical Sciences participated in the evaluation. Participants were selected using convenience sampling, while adhering to inclusion criteria (such as being enrolled for at least 2 semesters, absence of severe mental disorders, and no current use of psychiatric medications based on self-report). Exclusion criteria included instances like student expulsion or transfer to other educational institutions, rendering them inaccessible for the study (Mirhosseini et al., 2024).

### 2.6 Construct validity

Utilizing Maximum Likelihood Exploratory Factor Analysis (MLEFA) with Promax rotation, construct validity was initially assessed using data from 200 participants. Kaiser-Meyer-Olkin (KMO) and Bartlett tests were employed to assess sampling adequacy. KMO values between 0.7–0.8 and 0.8–0.9 were regarded as good and excellent, respectively. Items with factor loadings of approximately 0.33 or greater were deemed to belong to a latent factor, as estimated by the formula: CV = 5.152÷ √ (*n* – 2), where CV represents the number of extractable factors and n denotes the sample size. Subsequently, items with factor loadings below 0.3 were eliminated from the EFA analysis (Cattell, 1966; Cattell and Jaspers, 1967).

Next, Confirmatory Factor Analysis (CFA) was employed to validate the model derived from EFA (Kline, 2023). In essence, CFA assessed the goodness-of-fit of the proposed model against the actual model within the study population. Therefore, during this phase, the structural framework established through exploratory factor analysis underwent scrutiny via confirmatory factor analysis. Model fit indices including Root Mean Square Error of Approximation (RMSEA < 0.08), Comparative Fit Index (CFI > 0.9), Parsimony Comparative Fit Index (PCFI > 0.5), Parsimonious Normed Fit Index (PNFI < 0.5), Incremental Fit Index (IFI > 0.9), and (CMIN/DF > 3) were deemed acceptable (Hair et al., 2006; Bollen and Noble, 2011).

### 2.7 Convergent and discriminant validity

Convergent and discriminant validity of the RSES were examined following Fornell and Larcker’s approach, utilizing Average Variance Extracted (AVE), Maximum Shared Squared Variance (MSV), and Composite Reliability (CR) (Fornell and Larcker, 1981). Convergent validity is indicated by an AVE value exceeding 0.5 or a CR value surpassing 0.7. Moreover, confirming discriminant validity requires that the AVE exceeds the MSV value (Henseler et al., 2016).

### 2.8 Reliability

Cronbach’s alpha coefficient and McDonald’s omega coefficient were computed for each factor identified in the EFA to assess internal consistency. A minimum acceptable value of 0.7 was set for these coefficients, with higher values indicating favorable internal consistency within the factors. Furthermore, construct reliability (CR) was evaluated for each factor, with CR values exceeding 0.7 indicating good reliability (Mayers, 2013). External reliability, or the stability of the RSES, was evaluated using Intra-class Correlation Coefficients (ICC). As per the literature review, a minimum acceptable level for this index is 0.75 (Cicchetti, 1994). To assess this, a cohort of 40 medical sciences students completed the RSES twice, with a two-week interval between administrations.

### 2.9 Normality, outliers and missing data

Distribution diagrams and Mahalanobis distance (*p* < 0.001) were utilized to assess univariate and multivariate outliers. Additionally, skewness (values within ±3), kurtosis (values within ±7), and a Mardia coefficient < 8 were considered to investigate both univariate and multivariate normality distributions (Yuan et al., 2004; Mikkonen et al., 2022). Notably, in this study, the data did not significantly deviate from normal distribution. For estimating CFA, a listwise missing procedure was employed. Listwise deletion was favored over imputation due to non-response being linked with incomplete questionnaires. Statistical analysis was conducted using SPSS and Amos version 26.0.

### 2.10 Ethical approval and consent to participate

The Ethics Council in Biomedical Research of Semnan University of Medical Sciences approved this study (IR.SEMUMS.REC.1403.018). At the beginning of the research, participants were fully informed about the study’s objectives and the conditions for their participation. The authors adhered to the principles set forth by the Committee on Publication Ethics (COPE) in disseminating their findings. Written informed consent was obtained from all participants.

## 3 Results

The current study included a total of 380 medical sciences students. More than half of the participants (55.0%) were male, and about a fifth (22.4%) were studying medicine. The average age of the students was 22.49 ± 2.72 years. Table 1 displays the characteristics of the participants.

**Table 1:** The characteristics of medical science students (*n* = 380).

Variables		Number (%)
Gender	Male	209 (55.0)
	Female	171 (45.0)
Marital status	Single	354 (93.2)
	Married	25 (6.6)
	Divorced	1 (0.3)
Residence status	Student dormitory	239 (62.9)
	Rental house	15 (3.9)
	With family	126 (33.2)
		**Mean (SD)**
Age (years)		22.49 (2.72)
Interest in field (up to 10)		7.53 (1.99)

n, Frequency; SD, Standard deviation.

### 3.1 Face and content validity

Based on the findings of the face validity assessment, it was revealed that all items of the RSES were deemed appropriate, clear, and important, as indicated by quantitative face validity scores exceeding 1.5. Regarding qualitative content validity, revisions were made to some items based on feedback from 12 experts. For quantitative content validity, the Content Validity Ratio (CVR) and Content Validity Index (CVI) were calculated for each item individually, and none were deemed necessary for deletion, given the established cut-off point of 0.56. Furthermore, the modified Kappa statistic for all items surpassed the acceptable threshold of 0.7, indicating a satisfactory level of agreement.

### 3.2 Construct validity

In the exploratory factor analysis conducted with MLEFA, the Kaiser-Meyer-Olkin (KMO) measure yielded a value of 0.904, and Bartlett’s test of sphericity resulted in a statistic of 940.630 (*p* < 0.001). Employing Promax rotation, the model revealed two factors with eigenvalues surpassing one, collectively explaining 50.7% of the total variance (Sharif-Nia et al., 2024). Notably, no items were removed from the scale during this phase (Table 2).

**Table 2:** Exploratory factors analysis of the RSES (*N* = 190).

Factors	Qn. Item	Factor loading	h^2^	λ	%Variance
Positive	7: I am generally satisfied with myself	0.766	0.636	2.57	25.7
	6: I have a positive attitude toward myself	0.757	0.671		
	1: I feel that I am a valuable person (at least the same value as others)	0.741	0.504		
	2: I feel that I have good qualities	0.680	0.427		
	4: I can do things as well as other people	0.632	0.428		
Negative	9: Sometimes I feel useless	0.914	0.711	2.50	25.0
	10: Sometimes I think that I have no skills in any fields	0.764	0.606		
	3: All things considered, I usually feel like a failure	0.645	0.636		
	8: I wish I could have more respect for myself	0.622	0.356		
	5: I feel like I do not have much to be proud of	0.476	0.435		

h^2^, Item communalities; λ, Eigenvalue.

### 3.3 Confirmatory factor analysis

**Figure 1:**
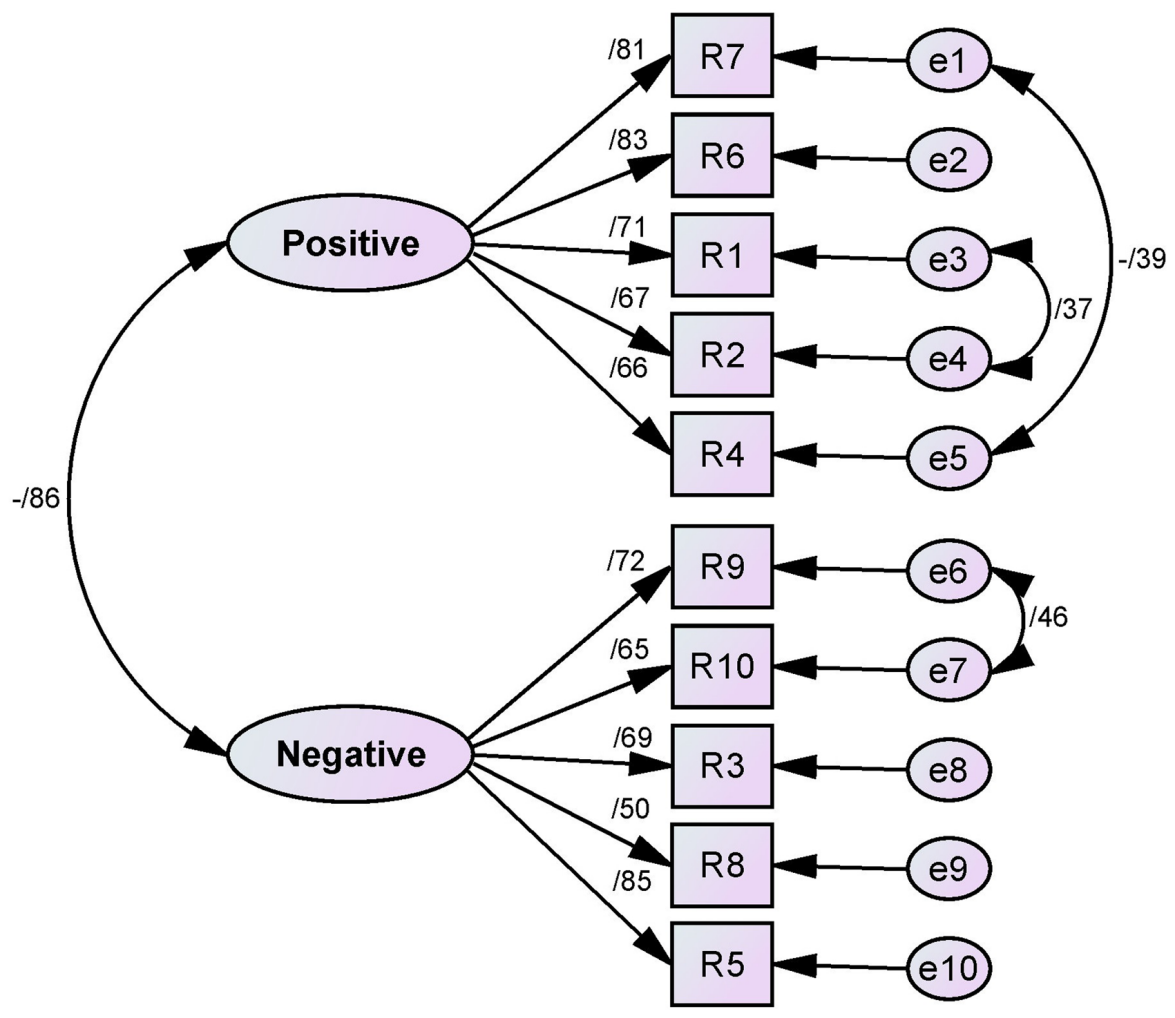
The final model of the RSES based on CFA (*N* = 190).

The findings from the CFA validated the final model, as all goodness-of-fit indices indicated satisfactory fit (χ^2^ = 54.660; DF = 31, *p* < 0.001, CMIN/DF = 1.763, PCFI = 0.671, PNFI = 0.649, RMSEA = 0.065, IFI = 0.974, CFI = 0.974, GFI = 0.944, and AGFI = 0.901) (Figure 1).

### 3.4 Convergent and discriminant validity

In terms of convergent validity, only Factor 1 (Positive Self-Esteem) had an Average Variance Extracted (AVE) greater than 0.5, indicating strong convergent validity. However, Factor 2 (Negative Self-Esteem) had an AVE of 0.475, slightly below the threshold. AVE is a reliable measure of convergent validity, but it’s worth noting that a Composite Reliability (CR) greater than 0.7 can independently assess convergent validity in psychological studies. Therefore, based on the CR and Maximum Shared Squared Variance (MSV) results, convergent validity was established for both factors. However, none of the factors demonstrated discriminant validity, as AVE was lower than MSV in all cases (Table 3).

**Table 3:** Convergent and discriminant validity, and reliability of the RSES.

Factors	CR	AVE	MSV	MaxR (H)	α	Ω	ICC
Positive	0/857	0/547	0/741	0/870	0.856	0.861	0.881
Negative	0/815	0/475	0/741	0/848	0.842	0.845	0.835

RSES: Rosenberg Self-Esteem Scale; CR: Composite Reliability; AVE: Average Variance Extracted; MSV: Maximum Shared Squared Variance; α: Cronbach’s alpha; Ω: McDonald’s omega; ICC: Intraclass Correlation Coefficients.

### 3.5 Reliability

The Cronbach’s alpha, McDonald’s omega, and ICC values for the two factors derived from RSES were found to be satisfactory. The ICC for positive and negative factors were calculated as 0.881 (95% CI: 0.746–0.944) and 0.835 (95% CI: 0.646–0.923), respectively. Additionally, a Composite Reliability (CR) above 0.7 indicated the scale’s reliability (Table 3).

## 4 Discussion

This section discusses the findings related to the psychometric evaluation of the Persian version of the Rosenberg Self-Esteem Scale. In line with the study’s objectives and criteria, the participants were medical sciences students. The gender distribution was nearly equal between males and females, and the majority of participants were single and living in student dormitories.

Based on the findings of this study, the results of the exploratory factor analysis revealed that the RSES scale comprises two factors: positive self-esteem and negative self-esteem. Overall, 10 items from this scale explain 50.7% of the total variance. Developed in 1965 by Rosenberg, the RSES aims to measure feelings of acceptance and overall self-worth in adolescents. Initially, Rosenberg conceived of this tool as a single-factor scale (Rosenberg, 1965). However, recent research has highlighted the presence of two factors within the scale. Utilizing the factor analysis method, researchers have shown that the RSES exhibits a two-factor structure, encompassing positive and negative self-images. Specifically, five items with positive wording (items R1, R2, R4, R6, and R7) load onto a factor termed “positive self-esteem” (PSE), while five items with negative wording (items R3, R5, R8, R9, and R10) constitute another factor named “Negative Self-Esteem” (NSE) (Fromont et al., 2017; Syropoulou et al., 2021; Kielkiewicz et al., 2020). In Iran, Rajabi and Karjo (2012) found that the two-factor model provides a better fit than the one-factor model. Similarly, studies conducted by Jamil (2006) among high school students in Malaysia, Mimura and Griffiths (2007) among Japanese participants, and Galanou et al. (2014) among Greek students have all reported a two-factor structure for the RSES, consistent with the findings of the present study. However, it is important to note that the present study’s findings regarding the RSES present it as a one-dimensional scale. Other research, such as that conducted by Martín-Albo et al. (2007) among Spanish students, Gómez-Lugo et al. (2016) among Colombian and Spanish individuals, Urbán et al. (2014) among Hungarian teenagers, Wu (2008) among undergraduate students in Taiwan, and Amahazion (2021) among Eritrean youth, have similarly reported the RSES as a one-dimensional scale.

In the Persian version of the RSES, the first factor extracted was positive self-esteem, comprising five items, all expressing positivity mainly related to individuals’ positive feelings about themselves. This factor accounted for the highest percentage of self-esteem and underscored its significance in assessing self-worth. Kaplan and Pokorny (1969), as well as Goldsmith (1986), posited that the first factor mirrors an individual’s positive perspective in defense of prestige and dignity, while the second negative factor reflects self-deprecation and unfavorable self-perception.

Serretti et al. (2005) identified a two-factor structure of the RSES, comprising self-confidence and self-dissatisfaction, which aligns with the positive self-esteem factor observed in the present study. According to the findings of Rajabi and Karjo (2012), confirmed through confirmatory factor analysis, two factors of the RSES scale encompass positive language, labeled “personal competence” and “self-satisfaction”, corresponding to the “positive self-esteem” factor identified in this study. Positive self-esteem, as defined by Coopersmith, refers to a personal assessment of one’s competence, shaped by one’s attitudes toward oneself. Individuals with positive self-esteem perceive themselves as capable, successful, noteworthy, and deserving (Coopersmith, 1967).

In the current version, the second factor extracted was the negative self-esteem factor, comprising five items with negative expressions. These items predominantly reflect individuals’ negative sentiments toward themselves. As noted earlier, Serretti et al. (2005) identified a two-factor structure of self-confidence and self-dissatisfaction within the RSES, analogous to the negative self-esteem factor observed in this study. Similarly, Kaplan and Pokorny (1969) and Goldsmith (1986) asserted that the second negative factor denotes self-denigration and an unfavorable attitude toward one’s competence and likability. Put simply, individuals with negative self-views perceive themselves as unimportant, unlovable, and lacking in self-confidence (Coopersmith, 1967).

Based on the results of the current study, all fit indices in the confirmatory factor analysis fell within the acceptable range, indicating that the model aligned well with the data. The original version of the RSES scale, along with some related studies, did not undergo evaluation via confirmatory factor analysis (Jamil, 2006; Coopersmith and Rosenberg, 1965; Schmitt and Allik, 2005). Consistent with the present study’s findings, Syropoulou et al. (2021) demonstrated that the model exhibited a good fit according to confirmatory factor analysis. Additionally, in their analysis to validate the Arabic version of the RSES, Ben Ayad et al. (2024) found that the model achieved a good fit with two factors.

According to the results of the present study, the RSES factors in the final model demonstrated good convergent validity, although discriminant validity was not confirmed for any of the factors. The original version of the RSES was not assessed in this regard (Coopersmith and Rosenberg, 1965). In a similar study conducted by Syropoulou et al. (2021), the convergent and discriminant validity of the RSES was evaluated using Fornell and Larcker’s approach, which involves calculating AVE, MSV, and CR. The findings indicated that the scale exhibited good convergent and discriminant validity (Syropoulou et al., 2021). Additionally, in a study by Eklund et al. (2018), it was found that the Swedish version of the RSES demonstrated both convergent and discriminant validity.

In the present study, both Cronbach’s alpha and McDonald’s omega coefficients for the two factors were higher than 0.7, indicating desirable internal consistency within the items of the Persian version of the RSES scale. Additionally, the Composite Reliability (CR) of the scale was assessed through confirmatory factor analysis (CFA), with all factors yielding values above 0.7, signifying the appropriate reliability of the structure. Consistent with the present findings, Rosenberg’s (1965) study on the RSES (Coopersmith and Rosenberg, 1965) and Vasconcelos-Raposo et al. (2012) reported Cronbach’s alpha coefficients exceeding 0.7 for two factors. Similarly, in a study by Li et al. (2019), Cronbach’s alpha values were 0.77 for the first factor and 0.62 for the second factor. Furthermore, in Amahazion’s (2021) study, the Cronbach’s alpha coefficient for the RSES was reported as 0.82, indicating excellent reliability. In line with these findings, Syropoulou et al. (2021) reported Composite Reliability (CR) values of 0.76 and 0.77 for the two positive and negative factors, respectively, indicating the appropriate reliability of the structure. Additionally, Boduszek et al. (2013) reported CR values deemed suitable for the two factors in their study.

The stability of RSES measurements was also assessed through test–retest analysis. The results revealed a significant correlation between the evaluations conducted at the first and second stages. This finding confirms the good reproducibility of the scale and demonstrates that the Persian version of the RSES scale exhibits acceptable stability, as indicated by the Intraclass Correlation Coefficient (ICC). Notably, the stability of this scale was not reported in its original version (Coopersmith and Rosenberg, 1965). In a similar study by Tulachan et al. (2022), the ICC values of the RSES scale ranged between 0.5 and 0.75 for each item, indicating moderate reliability, except for three items that exhibited poor reliability. In contrast to this study, Akhter and Ferdous (2019) as well as Rizwan et al. (2017) reported ICC results indicating moderate to good reliability across all items.

## 5 Conclusion

Overall, based on the findings, the total score of the Persian version of RSES ranges from 10 to 40, with higher scores indicating higher self-esteem among students of medical sciences. This scale comprises two factors: positive self-esteem, consisting of five items (with scores ranging from five to 20), and negative self-esteem, also comprising five items (with scores ranging from 5 to 20). This scale accounted for over half of the variance in students’ self-esteem, demonstrating its predictive power. Due to its user-friendliness, brevity, and comprehensibility, the RSES may serve as a practical and valuable tool for both research and clinical applications in Iran.

Since the participants were exclusively from the academic environment, generalizing the findings to the broader population may pose challenges. Hence, it is recommended to explore the psychometric properties of RSES at the community level. Additionally, there might be a common self-report response bias inherent in self-report instruments (Giromini et al., 2022), potentially impacting the quality of measurements due to inaccuracies in data entry or completion of the scale. Additionally, it should be noted that the sampling for this study was done using a convenience approach, with a significant number of participants being dormitory residents. As a result, the external validity of the study may be limited. For future research, we recommend using random sampling methods with greater precision to enhance validity. The results obtained are specific to Iranian culture. Therefore, it is recommended to evaluate the psychometric properties of the RSES in medical sciences students from other cultures.

## Data Availability

The original contributions presented in the study are included in the article/supplementary material, further inquiries can be directed to the corresponding author.
